# Letter Form as a Constraint for Errors in Neglect Dyslexia and Letter Position Dyslexia

**DOI:** 10.1155/2005/635634

**Published:** 2006-01-09

**Authors:** Naama Friedmann, Aviah Gvion

**Affiliations:** ^1^Tel Aviv UniversityTel AvivIsrael; ^2^Reuth Medical CenterTel AvivIsrael

## Abstract

Does letter-form constrain errors in peripheral dyslexia? In Hebrew, 5 of the 22 letters have two different letter forms, one is used only when the letter occurs in word-final position, the other form is used in initial and middle positions. Is the information on final-forms encoded in the letter identity information and used for word identification, or is it discarded? The current research explored this question through the effect of final vs. non final letter form on the error pattern in neglect dyslexia (neglexia) and letter position dyslexia (LPD). Left word-based neglexia results in errors of omission, substitution and addition of letters in the left side of words, which in Hebrew is the end of the word. We examined whether final letter form blocks the addition of letters to the end of the word and whether omissions of letters after letters in non-final form are avoided. The predominant error type in LPD is migration of letters within words. We tested whether migrations also occur when they cause form change of either final-form letters that move to middle position or middle-form letters that move to final position. These questions were assessed in both acquired and developmental neglexia and LPD. The results indicated a strong effect of final letter-form on acquired neglexia and on acquired and developmental LPD, which almost completely prevented form-changing errors. This effect was not found in developmental neglexia, where words that end in final-form letters were actually more impaired than other words, probably because final-form letters appear only on the neglected side of the word for Hebrew-reading children with left developmental neglexia. These data show that early visuo-orthographic analysis is sensitive to final letter form and that final letter form constrains errors in peripheral dyslexia.

## 1. Introduction

The first stage of word reading includes the extraction of letter identities from the written word. This requires abstracting away from the size of the letters, their case, font, beauty of handwriting and many other aspects of the written letters [1–3,6]. The current study asked whether we also ignore letter forms that reflect, in addition to letter identity, also letter position, or whether such letter forms are encoded. This question was explored through the examination of whether this type of information modulates errors in peripheral dyslexias.

The study used a special characteristic of Hebrew orthography to assess this question: Hebrew has 22 letters, five of them occur in two different forms – one form is used for word-initial and medial positions, the other is used only when the letter is located in final position; the other 17 letters maintain the same form across all letter positions. See (1) for the Hebrew alphabet, and (2) for the letters that occur in two forms – in each pair the initial/medial form is on the right and the corresponding final form on the left. Example (3) presents two words that compose only the letters /m/ – which appears as מ in initial and middle position and ם in final position, and the letter /c/ – צ in initial and middle position and ץ in final position, one word is read "cimcem" and means "reduced", the other word reads "micmec" and means "blinked" – these words show the use of the same letter in the two forms.

(1) א ב ג ד ה ו ז ח ט י כ ל מ נ ס ע פ צ ק ר ש ת

(2) נן מם כך צץ פף

ISSN 0953-4180/05/$17.00 © 2005 – IOS Press and the authors. All rights reserved

(3) צמצם מצמץ

Another relevant property of Hebrew is that it is read from right to left. This direction of reading causes left word-based neglexia to impair final letters, rather than first letters as is usually the case in left-to-right orthographies. These two properties of the Hebrew orthography – final form letters and the direction of reading – allow the exploration of the question of the encoding of the letter form information, as well as questions regarding the interrelations between final letter form and errors in dyslexia.

Do readers abstract away from letter form when identifying a final-form letter, or do they encode and use the information about location from the final form? If they encode this information – how do they encode it?

Three parallel sources of information have been proposed to be involved in word recognition, in the first stages of visual-orthographic processing: Abstract letter identity (ALI), holistic word-shape, and multi-letter units [10,12]. Before discussing ALI, let us just briefly explain why final-forms in Hebrew cannot be encoded as part of the word-shape information, or as part of the multi letter units. It cannot be part of the holistic word-shape, because though four of the Hebrew final letters do have a distinctive contour, there is one final letter with a non-distinctive contour and one non-final letter with a contour similar to that of the extending final letters, so they cannot be part of the word-shape encoding. In addition, they cannot be considered part of the transletter features or multi-letter units, which specify the visual relations between frequent, common adjacent letters or letter groups, particularly those that might act as graphemic units [10,12]. This is because unlike morphemes and common letter groups, the appearance of final letters is not predicted from the environment of letters.

One type of encoding which can host information about final forms is the abstract letter identity, ALI [1–3,6,15,18,21] but see [13] findings and review on the effect of case mixing on word familiarity; see [19] for a review]. The creation of abstract letter identity representations involves abstracting away from information that is irrelevant to word reading, such as information about fonts, size, case, or letter color.

The question now is whether final-form is encoded (as part of the ALI or as an additional source of information), or whether it is discarded. If letter form is encoded during the early stages of Hebrew word reading, then letter form can affect reading and modulate errors in peripheral dyslexias. Two peripheral dyslexias can shed light on these questions: word-based neglect dyslexia (neglexia) and Letter position dyslexia (LPD).

Neglexia is a reading deficit that involves a failure to attend to one side of the word or text, usually to the left side [4,5,11,14,20]. Word-based neglect dyslexia impairs the ability to identify or report letters on one of the sides of the word, resulting in substitutions, omissions or additions of letters on that side. Usually it is the left side that is impaired, and therefore initial letters are neglected in left-to-right languages [4,5,11,14,20]. In right-to-left languages like Hebrew, final letters are impaired [8,17]. If the information about final letter form is used in reading, and encoded in some way in the initial orthographic-visual representation of the written word (either within the ALI or in a different type of encoding), then it might be used to signal the end of the word for individuals with neglexia, and therefore to prevent additions following a final-form letter. The existence of two letter forms might also indicate that when reading a letter that is in the middle form, this cannot be the end of the word, and the reading should proceed further to the left. Therefore, the questions that we will address in the analyses of the effect of final forms on reading in neglexia will be whether additions of letters at the end of the word occur less frequently after a final-form letter than after a regular-form letter, and whether omissions of final letters occur less frequently when the resulting word is a word that ends in a form-changing letter that is in the wrong (middle) letter-form. The sensitivity to final form letter will be also investigated by examining whether, given two options for a neglexia error that results in a lexical item, non-form-changing errors are preferred over form-changing errors. Finally, we will examine whether there are less neglect errors in words that end in final-form compared to the rest of the words.

Another dyslexia that might show effects of letter-form at the orthographic-visual analysis stage is letter position dyslexia (LPD), a dyslexia that is caused by a selective failure to encode letter positions within words. This dyslexia was detected in both acquired and developmental forms [7,9]. The predominant error type in LPD is migration of letters within words, like reading ‘beard’ instead of ‘bread’, without letter identity errors. This would allow us to ask whether, when letter position encoding is impaired, letter form that encodes position can be used. This would be explored by testing whether final letter-form blocks the movement of a final letter in final-form to a middle position in the word, and similarly, whether it blocks the migration of form-changing middle letters to final position.

(This comparison will relate to errors that involve final letters, although most migration errors in LPD are of medial letters within words, with very few migrations involving exterior letters).

In neglexia and LPD, both acquired and developmental cases were reported. The acquired and the developmental cases consistently manifest similar symptoms. However, unlike individuals with acquired dyslexia, who have established the mechanisms that relate to the visual analyzer prior to the onset of dyslexia, in developmental dyslexia the deficit is present throughout the acquisition of reading. Will this cause difference in the ability to encode final letter forms?

The current study analyzed the sensitivity to final letter form in the single word reading of 33 individuals with dyslexia. Sixteen individuals with left word-based neglexia – 7 with acquired neglexia and 9 with developmental neglexia, and 17 individuals with LPD – 2 with acquired LPD and 15 with developmental LPD.

In the next section detailed research questions, method and results on acquired and developmental neglexia will be presented, followed by a section on research questions, method and results on acquired and developmental LPD.

## 2. Experimental investigation: Acquired and developmental neglexia

In order to explore the effect of letter form on neglexia, we assessed the effect of final letter form on neglect errors in 16 Hebrew-speaking individuals with left word-based neglexia: 7 with acquired neglexia and 9 with developmental neglexia.

We focused on four main questions: The first was whether additions of letters at the end of the word occur less frequently after final-form letter, which signals the end of the word. For this analysis we compared the number of letter additions after final form-changing-letters that appear in their final form to additions after final letters that do not change their form.

Example (4) shows a word that ends with a final-form letter. Notice, that addition after the final letter requires a change in the letter form because the letter is no longer final. Example (5) shows an addition after a letter that does not change its form. (All the examples in this article will be presented in the following order: Hebrew word, orthographic transcription in the same order of letters (leftmost letter in Hebrew is also leftmost in the transcript) with final-form letters in capital, phonological transliteration, and a translation).

(4) חוף (Fox, xof, beach) ⇨ חופר (rfox, xofer, digs)

(5) חוק (kox, xok, law) ⇨ חוקר (rkox, xoker, researcher)

The second question focused on the opposite direction: whether omissions of final letters occur less frequently when the resulting word is a word that ends in a form-changing letter that is in the wrong (middle) letter-form. For this analysis we compared omissions of final letters after form-changing-letters to omissions of letters that follow non-form-changing-letters. Example (6) shows a word with a penultimate form-changing letter, which appears in middle form. The omission of the final letter that follows it requires change of the form, because now it became the final letter and has to appear in final-form. Example (7) shows a similar omission, but this time after a penultimate letter that does not change its form.

(6) חופר (rfox, xofer, digs) ⇨ חוף (Fox, xof, beach)

(7) חוקר (rkox, xoker, researcher) ⇨ חוק (kox, xok, law)

In the analyses of reading in acquired neglexia, we included only words for which the relevant error creates a lexical response. Namely, for the analysis of additions we compared only words with a lexical potential for addition with and without final-form letter in final position. For the analysis of omissions we compared only words that create another existing word following an omission error, with or without a form-changing letter in penultimate position. In the analyses of the developmental neglexia we did not calculate lexical potential, but included all words, because lexicality did not play a role in these children's reading, and they frequently produced non-lexical responses.

The third analysis looked at words that can be read either with a final-form-violating error or with another error that does not violate final form, and analyzed whether there was a preference to make non-form-changing errors. This was again done both for additions and for omissions. For additions, we looked at words that had a potential for either addition after a final form letter or substitution or omission; for omissions we looked at omissions after a penultimate form-changing letter compared to substitutions and additions. This analysis could only be carried out for the acquired cases because there was no point in calculating potentials for errors given the production of nonwords in the developmental cases.

The final question in both populations was whether the general error rate in words that end in final-form letters is different from that of the rest of the words. This comparison was aimed at finding out whether the existence of a final-form letter in general modulates the general rate of correct reading.

### *2.1. General procedure*

In all the experiments reported in this article (on acquired and developmental neglexia and LPD), each of the participants was tested in a quiet room, they were instructed to ask for a break or to terminate the session altogether whenever they felt tired. No time limit was imposed during testing, and no response-contingent feedback was given by the experimenter. The number of words that were completed from the word lists varied between participants, so all tables in this article include the total number of relevant words for each participant in addition to the percentage of errors. Words were printed in Arial font on a page, each word was presented separately. Each participant saw each word only once. All words in this research were presented without diacritics.[1] Words were chosen according to the type of final and medial letters, and the lexicality of the result of omissions, additions and final letter migration errors. Only words that allow for a lexical result following the relevant errors type (neglect errors in the lists for the individuals with neglect, letter migration errors in the lists for the individuals with LPD) were included in the analyses. Some of the children with the developmental dyslexias had also surface-dyslexia-like errors, which resulted from an incomplete lexicon. These errors were not counted as errors in our analyses. Error rate and error type analysis were done for each individual participant and for each group, by 3 independent judges. There was agreement on all classifications but 3 words, which were then discussed and their analysis was agreed upon by the judges. Only first responses were analyzed. Paired analyses within each group were done using Wilcoxon signed rank test and an alpha level of 0.05.

[1] Hebrew has two orthographic systems. In the common system, which is used in books, newspapers, and signs, vowels are usually not represented in the orthography, and many written words comprise only consonant letters. In addition to this system, another orthographic system exists, in which vowels (as well as some information about consonants) are represented by diacritics – small lines and dots above, below and inside letters. Diacritics are used in children's books, prayer books, and poetry.

### *2.2. Acquired neglexia*

#### *2.2.1. Participants*

The participants were 7 adults with acquired word-based neglexia, aged 43–79, 3 women and 4 men. They all had a brain lesion following stroke in the right hemisphere that caused neglect dyslexia. They were all speakers and readers of Hebrew, without premorbid reports of developmental dyslexia. Their details are given in the Appendix. For all the participants, neglect errors were the predominant error type (93% of the errors), and less than 1% of their responses were errors other than neglect errors. They made 15%–57% neglect errors in reading ($M = 26\%$). Their neglect errors were substitutions, additions and omissions, these were manifested by all participants, except BT who did not have omissions.

#### *2.2.2. Results*

The results indicated that errors in acquired neglexia are sensitive to letter form and almost never create a word that is ill-formed, that contains final-form letter in middle position or middle-form letter in final position.

Table 1 presents the results of the analysis of addition errors in words that end in final form letter vs. other words. Table 2 presents the results of the analysis of omission errors after a form-changing middle letter vs. other letters. The statistical analyses revealed that there were significantly less additions of letters on the leftmost side after a final-form letter than after other letters, $T(7) = 0$, $p = 0.008$, and that there were significantly less omission errors after a form-changing letter in penultimate position than after other letters, $T(6) = 0, p = 0.016$.

The next analysis looked at words that had more than one possible lexical error, one violating final form requirements and at least one other that does not violate letter form. This analysis showed that while there was no clear preference for omission or addition when the words did not include a final-form letter, there was a clear refraining from omission and addition when they caused violation of letter form, and preference for the other error type when it allowed avoiding form-change, see Table 3 for additions vs. other errors and Table 4 for omissions vs. other errors (Participant MH was excluded from this type of analysis because he produced many non-lexical responses and therefore we could not do the analysis of lexical potentials for him). In words that allowed both lexical additions and lexical substitutions or omissions, the rate of addition errors to other errors was significantly lower in words in which

Table 1
Additions after final-form letter vs. additions after other letter

| Participant | Final form letter | | Other letter | |
|---|---|---|---|---|
| | % errors | errors/total | % errors | errors/total |
| BR | 4% | 1/26 | 21% | 8/39 |
| HZ | 0% | 0/17 | 11% | 3/27 |
| ZR | 0% | 0/251 | 20% | 6/30 |
| XM | 0% | 0/14 | 14% | 6/42 |
| BT | 0% | 0/22 | 4% | 2/56 |
| CL | 0% | 0/17 | 6% | 3/49 |
| MH | 0% | 0/19 | 11% | 5/46 |
| **Total** | **1%** | **1 /136** | **11%** | **33 /289** |

Table 2
Omissions after penultimate form-changing letter vs. omissions after other letters

| Participant | Form-changing Penultimate letter | | Other Penultimate letter | |
|---|---|---|---|---|
| | % errors | errors/total | % errors | errors/total |
| BR | 0% | 0/19 | 10% | 7/68 |
| HZ | 13% | 2/16 | 16% | 8/49 |
| ZR | 0% | 0/11 | 28% | 13/46 |
| XM | 0% | 0/20 | 21% | 12/58 |
| CL | 0% | 0/25 | 3% | 2/63 |
| MH | 4% | 1/26 | 16% | 12/73 |
| **Total** | **3%** | **3 /117** | **15%** | **54 /357** |

the addition violated letter-form than in words in which additions did not change letter form, $T(6) = 0$, $p = 0.016$. The same held for omissions: significantly lower omission-to-other errors rate when the omission violated final letter form, $T(5) = 0$, $p = 0.03$. An interesting example is the reading of XM, who has a clear preference for omission errors (making almost 3 times more omissions than other neglect errors) – in words in which omission causes a form change he made no omissions at all. These results indicate that when given several lexical error options, final letter form mediates the "choice" of error, and errors that cause violations of letter form are hardly ever produced. A different way to look at it is that errors that violate letter form are not a possible error type.

The final analysis was aimed at finding out whether words that end with final-form letters are more or less prone to error compared to words that end in other letters. Only words with a potential for a lexical response following a neglect error (of any type) were included in this analysis. The results of this comparison are given in Table 5. Words with final-form letters in final position were read significantly better than other words, $T(6) = 1$, $p = 0.03$. On the individual level, this comparison was significant for 3 of the 6 participants.

One supplementary analysis was done in order to find out whether the visual saliency of some of the final-form letters was responsible for the difference in reading performance between words with and without final forms, as 4 of the 5 final form letters extend below the line of writing (see Example (2)). We compared the reading of words that end with extending final form letters "ך, ץ, ף, ן", to words that end in the non-extender final form letter "ם", and words that end with non-final non-extending letters to the non-final yet extending "ק". The reading of words with extending final-form letters did not differ from that of words that end in a non-extending final-form letter, $T(6) = 3, p = 0.16$. Moreover, no difference in the number of neglect errors was found between words that end in extending and non-extending non-final letters, $T(6) = 10, p = 0.5$. This indicates that it is *not* the extension below the line that made words with final-form letters less prone to errors in acquired neglexia.

### 2.3. *Developmental neglexia*

The next step was to assess whether the effect of final letter-form that we found in acquired neglexia applies also to children with *developmental* neglexia. Because the five final-form letters appear only in the left side of words, they are constantly in the neglected side of the children with neglexia. Unlike the individuals who acquired neglexia after they had established a stable representation of final letters, children whose reading acquisition is marred by neglexia might actually have more difficulty in reading final letters, because they were never able to attend normally to final letters that

Table 3
Type of error produced in words that allow both addition and other error types (omission/substitution): comparison of avoidance of additions when they change and do not change letter form

| Participant | Non final form | | | | Final form | | | |
|---|---|---|---|---|---|---|---|---|
| | Non form-changing addition | | Substitution/ omission | | Form-changing addition | | Substitution/ omission | |
| | %errors | errors/total | %errors | errors/total | %errors | errors/total | %errors | errors/total |
| BR | 15% | 6/39 | 10% | 4/39 | 4% | 1/26 | 12% | 3/26 |
| HZ | 10% | 3/29 | 21% | 6/29 | 0% | 0/19 | 11% | 2/19 |
| ZR | 25% | 9/36 | 33% | 12/36 | 0% | 0/25 | 32% | 8/25 |
| XM | 13% | 6/48 | 13% | 6/48 | 0% | 0/15 | 0% | 0/15 |
| BT | 3% | 2/59 | 12% | 7/59 | 0% | 0/22 | 14% | 3/22 |
| CL | 10% | 5/50 | 6% | 3/50 | 0% | 0/17 | 6% | 1/17 |
| **Total** | **12%** | 31/261 | **15%** | 38/261 | **1%** | 1/124 | **14%** | 17/124 |

Table 4
Type of error produced in words that allow both omission and other error types (addition/substitution): comparison of avoidance of omissions when they change and do not change letter form

| Participant[1] | Non form-changing | | | | Form-changing | | | |
|---|---|---|---|---|---|---|---|---|
| | Non form-changing omission | | Substitution/ addition | | Form-changing omission | | Substitution/ addition | |
| | %error | errors/total | %error | errors/total | %error | errors/total | %error | errors/total |
| BR | 12% | 8/67 | 15% | 10/67 | 0% | 0/17 | 24% | 4/17 |
| HZ | 16% | 8/51 | 12% | 6/51 | 7% | 1/14 | 29% | 4/14 |
| ZR | 28% | 17/60 | 27% | 16/60 | 0% | 0/15 | 60% | 9/15 |
| XM | 18% | 11/60 | 7% | 4/60 | 0% | 0/17 | 24% | 4/17 |
| CL | 3% | 2/64 | 17% | 11/64 | 0% | 0/18 | 11% | 2/18 |
| **Total** | **12%** | **46/380** | **16%** | **59/380** | **1%** | **1/81** | **28%** | **23/81** |

[1] BT was not included in the analysis of omissions because she made no omission errors.

appear exclusively on the neglected side of words. On the other hand, they might improve reading because 4 of the 5 final-forms extend below the line of writing and might therefore attract attention to the left side of words.

### 2.3.1. *Participants*

The participants were 9 individuals with developmental left word-based neglect dyslexia. Their ages were 9;1–12;11 (mean age 10;8), they were between 3rd and 6th grade. All of them had only word-based neglect without general neglect or neglect at the text level. They were all native speakers of Hebrew and right handed, without known history of brain lesion, neurological disease or loss of consciousness. Their details are given in the Appendix. For all the participants, neglect errors were the predominant error type, which accounted for 78% of their errors. The other error type that most of them made in addition to neglexia errors were errors of reading via grapheme-to-phoneme conversion ("surface-dyslexia-like" errors) that probably resulted from their limited exposure to reading due to their reading difficulties. They made neglect errors on 29%–85% of the words ($M = 51\%$). Their neglect errors were mainly substitutions and omissions, which were manifested by all participants, and some of them also made addition errors.

### 2.3.2. *Results*

Unlike the individuals with acquired neglexia, the 9 children with developmental neglexia did not show effects of letter form and did not show better reading of words ending in final-form letters, probably because neglexia was present during their reading acquisition, preventing them from allocating attention to these letter-forms that always appear in their neglected side.

The children with developmental neglexia did not show any difference between additions after a final-form letter and after other letters, $T = 21, p = 0.91$; the analysis of the omissions yielded similar results, omissions did not occur less after penultimate final-form letter than after other letters for any of the participants, $T = 15, p = 0.74$, see Tables 6 and 7.

The analysis of the general number of errors in words ending with a final-form letter compared to words ending with other letters yielded a remarkable result: more errors were made in reading words with final-form letters than in other words (this difference was marginally

Table 5
Errors in reading words that end with final-form letters vs. words that end in other letters

| Participant | Final-form letter | | Other final letters | |
|---|---|---|---|---|
| | % errors | errors/total | % errors | errors/total |
| BR | 28% | 10/36 | 25% | 20/80 |
| HZ | 15% | 4/27 | 38% | 23/61 |
| ZR | 36% | 12/33 | 68% | 50/73 |
| XM | 4% | 1/27 | 29% | 28/97 |
| BT | 11% | 4/38 | 16% | 20/123 |
| CL | 12% | 4/33 | 18% | 19/103 |
| **Total** | **18%** | **35/194** | **30%** | **160/537** |

significant, $T(9) = 9$, $p = 0.06$), see Table 8. On the individual level, for 4 participants words with final-form letters were read significantly worse than other words, using $\chi^2$, $p < 0.05$, and for one participant it was marginally significant.

As can be seen in Fig. 1, this result forms an important difference between acquired and developmental neglexia: in acquired neglexia final-form letters reduced the total number of errors, whereas in developmental neglexia they increased the error rate. Two-factor ANOVA with repeated measures on word type showed a significant interaction between group (acquired/developmental) and words with and without final letter form, $F(1, 13) = 9.98, p = 0.007$.

Like in the analysis for the acquired neglexia, here, too, the fact that some final letters extend below the line did not help reading. Nachman-Katz [16] compared the reading of words that end with extending final form letters to the non-extending final form letter, and the reading of words that end in an extending non-final form letter to other non-final form letters. None of the participants showed a difference in total number of errors between words that end with regular letters that do not extend below the line and the non-final form "ק", which does extend below the line, this difference was also not significant on the group level, $T(9) = 14$, $p = 0.36$. Words with the final-form "ם", which is the only final form that does not extend below the line, were even read better than words with the other final-form letters that do extend below the line ("ך, ץ, ף, ן"), $T(9) = 3, p = 0.02$.

As reported by Nachman-Katz [16], eight of the participants with developmental neglexia had difficulties also in identification or naming of single final-form letters (but did not have difficulties with middle-form letters), and five of the six participants who also had neglect dysgraphia did not use final form letters in writing [17].

## 3. Experimental investigation: Acquired and developmental LPD

Letter position dyslexia (LPD) is a selective deficit in letter position encoding that results in errors of letter position within words. This deficit occurs both in acquired and in developmental form. In both cases, errors occur predominantly in "migratable" words, namely words for which letter migration creates another existing word. The vast majority of migration errors occur in middle positions: Friedmann and Gvion [7] reported two individuals with acquired LPD for whom middle letter migrations occurred 19.5 times more frequently than migrations that involved exterior letters. The same holds for developmental LPD: Friedmann and Rahamim (2004) reported 11 individuals with developmental LPD who had 24 times more migrations of adjacent middle letters than of exterior letters [7]. However, we can still analyze the (fewer) exterior migration errors with respect to whether or not they violate final letter form. Notice that when a final form letter in final position moves to a non-final position, it has to change its form – see example (8) for a form-changing exterior migration error, and example (9) for a similar exterior migration involving letters that do not change their form.

(8) חורף (Frox, xoref, winter) ↔ חופר (rfox, xofer, digs)

(9) חורק (krox, xorek, squeaks) ↔ חוקר (rkox, xoker, researcher)

Does final letter-form protect the five final form letters from migrating into a different position in the word? Namely, does letter-form prevent final letters from moving to a middle position, and middle letters from moving to final position?

### 3.1. Acquired LPD

Words for which a migration of exterior letters creates a lexical result (another existing word) were pre-

Table 6
Additions after final-form letter compared to other letters

| Participant | Final-form letter | | Other final letters | |
|---|---|---|---|---|
| | % errors | errors/total | % errors | errors/total |
| NT | 5% | 1/21 | 2% | 1/59 |
| IZ | 4% | 1/27 | 1% | 1/73 |
| VL | 3% | 1/39 | 2% | 3/153 |
| SP | 0% | 0/20 | 1% | 1/69 |
| ID | 3% | 1/36 | 10% | 13/124 |
| SS | 0% | 0/38 | 3% | 3/120 |
| OR | 0% | 0/26 | 6% | 5/84 |
| DN | 6% | 2/36 | 4% | 5/124 |
| ST | 3% | 1/30 | 0% | 0/98 |
| **Total** | **3%** | **7/273** | **4%** | **32/904** |

Table 7
Omissions after penultimate form-changing letter compared to omissions after other letters

| Participant | Form-changing penultimate letter | | Other penultimate letter | |
|---|---|---|---|---|
| | % errors | errors/total | % errors | errors/total |
| NT | 11% | 2/19 | 11% | 6/57 |
| IZ | 37% | 10/27 | 25% | 26/102 |
| VL | 10% | 5/49 | 5% | 7/132 |
| SP | 11% | 3/27 | 15% | 10/67 |
| ID | 0% | 0/37 | 1% | 1/102 |
| SS | 11% | 3/28 | 12% | 14/117 |
| OR | 4% | 2/50 | 7% | 6/91 |
| DN | 13% | 7/54 | 9% | 11/120 |
| ST | 18% | 4/22 | 17% | 16/93 |
| **Total** | **11%** | **36/313** | **11%** | **97/881** |

sented to two Hebrew-speaking men with LPD, BS and PY, 75 and 70 years old respectively, with no premorbid reading disorder (for further details see the Appendix, and [7]). They had 16% and 26% migration errors in reading respectively, most of these errors occurred in middle positions.

Words in which exterior letter migration causes a change in letter form were compared to words for which exterior letter migration does not change letter form. Together, in all word lists, BS and PY made only 6 errors of exterior letter migration. All of these 6 errors included only letters that do not change their letter form, and no error included a final-form letter.

### 3.2. *Developmental LPD*

The larger number of participants with developmental LPD allowed for a more detailed quantitative analysis of the effect of final letter form on letter migrations within words. It also allowed for the assessment of whether our earlier finding of the lack of final form letters effect in children with developmental neglexia resulted from the fact that they acquired reading while having dyslexia, or from the specific deficit that children with neglexia have with respect to letters that appear exclusively on the neglected left side of words.

For this analysis we presented the participants with words for which a migration of exterior letters creates a lexical result. Words in which exterior letter migration causes a change in letter form were compared to words for which exterior letter migration does not change letter form. Change in letter form was counted as migration of a form-changing letter both from final position and final form to middle position and middle form, and from middle position and middle form to final position and final form. The knowledge of the words (both of the target words and of the possible results of migration) of children in these ages was assessed by nine special education teachers who are well-experienced in working with children in these ages, and words that were judged as even possibly unfamiliar to participants at a certain age were excluded from the list for the relevant children before the analysis.

#### 3.2.1. *Participants*

Fifteen individuals with developmental LPD participated in this experiment. The reading pattern of 11 of them is reported in detail in Friedmann and Ra-

Table 8
Reading words that end with final-form letters vs. words that end in other letters

| Participant | Final-form letters | | Other final letters | |
|---|---|---|---|---|
| | % errors | errors/total | %errors | errors/total |
| NT | 66% | 23/35 | 45% | 67/148 |
| IZ | 71% | 32/45 | 51% | 99/195 |
| VL | 47% | 21/45 | 25% | 48/195 |
| SP | 97% | 29/30 | 82% | 127/154 |
| ID | 71% | 35/49 | 58% | 132/226 |
| SS | 33% | 10/30 | 49% | 56/114 |
| OR | 41% | 9/22 | 42% | 33/78 |
| ST | 30% | 8/27 | 40% | 30/74 |
| DN | 52% | 14/27 | 38% | 14/37 |
| **Total** | **58%** | **81/310** | **50%** | **606/1221** |

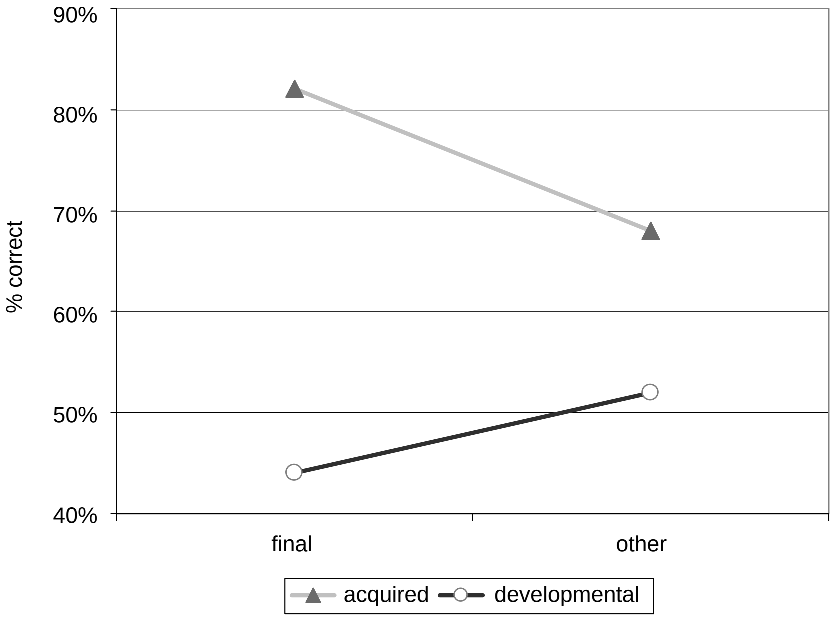

Fig. 1. The effect of final form letters on reading in acquired and developmental neglexia.

hamim [9]. They were 14 children and adolescents aged 9;4–15;9 (mean age 11;4) and one adult aged 29;10. See the Appendix for their background details. For all of them the predominant error in reading was (middle) letter migration, their migration error rate ranged between 14% and 45% ($M = 25.5\%$).

*3.2.2. Results*

Similarly to the adults with acquired LPD, the individuals with developmental LPD were significantly less prone to migration errors that caused change of letter form when the final letter became non-final (or when the penultimate letter became final).

In general, in the same vein with previous findings about LPD [7,9], there were relatively few exterior letter migrations. While these participants made an average of 25% errors of middle letter migrations, they made only 3% migrations that involved exterior letters. However, because of the large number of items and participants, we were able to collect 116 exterior letter migration errors and analyze them. Out of these 116 errors, only 6 involved letters in final forms, 5 of them produced by the same participant, SP, see Table 9. These 6 errors were only 0.2% of the words that had a lexical potential for migration of final-form letters. This difference between exterior migrations that involve final-form letters and exterior migrations that involve other letters was significant for the group, $T(14) = 0$, $p < 0.0001$. The same pattern held for each of the 14 individual participants that made any exterior letter migrations, and was statistically significant

Table 9
Exterior migrations in developmental LPD: migrations that involve final-form letters vs. migrations of other letters

| Participant | Final-form letters | | Other letters | |
|---|---|---|---|---|
| | % errors | errors/total | % errors | errors/total |
| SP | 3% | 5/200 | 5% | 21/421 |
| YS | 0% | 0/172 | 3% | 10/319 |
| DV | 0% | 0/139 | 1% | 2/286 |
| HN | 0% | 0/139 | 7% | 19/286 |
| SL | 0% | 0/139 | 2% | 7/286 |
| SN | 1% | 1/139 | 2% | 6/286 |
| NS | 0% | 0/139 | 1% | 3/286 |
| RM | 0% | 0/139 | 1% | 3/286 |
| RI | 0% | 0/139 | 4% | 11/286 |
| AN | 0% | 0/139 | 3% | 8/286 |
| HA | 0% | 0/70 | 7% | 9/126 |
| ST | 0% | 0/61 | 2% | 3/135 |
| SR | 0% | 0/55 | 0% | 0/121 |
| RMI | 0% | 0/49 | 5% | 5/106 |
| SNI | 0% | 0/49 | 3% | 3/106 |
| **Total** | **0.2%** | **6/1768** | **3.0%** | **110/3622** |

for five of them (using $\chi^2$, $p < 0.05$, and marginally significant for another, $p = 0.06$).

## 4. Discussion

This study explored the sensitivity of the visual analyzer to final letter form by assessing the effect of final form on errors in two peripheral dyslexias: word-based left neglect dyslexia and letter position dyslexia. In Hebrew, 5 of the 22 letters appear in different forms in middle and initial word position compared to final position, which in Hebrew, a language read from right to left, is the left side of the word. Two main comparisons were conducted. One comparison was of number and type of errors in reading words with and without form-changing letters. The other analysis compared acquired and developmental forms of the same dyslexia with respect to the sensitivity to form-changing letters.

The main findings were that individuals with acquired word-based neglect dyslexia hardly ever make errors that violate the letter form – they do not produce responses that cause a form change in letters – they do not add letters after a letter in final-form (which would require it to change its form to non-final), and they do not omit letters after a letter in non-final form, which would leave it in non-final form, but in final position. When a word is susceptible to more than one error type that yields a lexical response, one error violating letter-form and the other does not violate letter form, they refrain from producing the error that violates letter form, and if they make an error, they produce the non-violating error. They make less errors in general on words that end in final-form letters, probably because this final form is used as an additional information regarding the left side of the word.

Similar results were found with respect to errors in LPD. The main characteristic of LPD is letter migrations within words. Most of the migrations are of middle letters, but migrations that involve final letter and penultimate letter also occur, to a lesser extent. The analysis of migrations that involve the final letter showed that individuals with acquired and developmental LPD almost do not make letter order errors that change letter-form, and therefore final-form letters remain safe in their word-final position, and non-final form letters do not move to final position.

Unlike individuals with acquired neglexia and individuals with acquired and *developmental* LPD, children with developmental neglexia cannot use final-forms as a cue for the left side of the word, and the reading of 4 of them was even significantly poorer in words that end with the final-form letters. This can be explained by the fact that these final-form letters appear only in the neglected side of words, and given that their neglexia was present throughout the process of reading acquisition, they were never able to attend normally to these letters, and therefore they could not establish normal encoding of these final-form letters (either as part of the abstract letter identification unit or as a separate encoding). This, in turn, led to their special difficulty in reading final-form letters. This difficulty is a specific result of developmental neglect dyslexia and is *not* a general property of developmental dyslexia, as can be seen from the finding that children with developmental

LPD were sensitive to final letters, and almost never made form-changing errors.

What is the mechanism through which letter-form modulates neglect errors in acquired neglexia and in LPD? It might be that final-form letters play an attentional role, and when the visual analyzer identifies a form-changing letter that is not in final form, it serves as a signal for the orthographic attentional processes to proceed in allocating attention leftwards, with a result of significantly less omissions of letters following the form-changing letter. The identification of final-form letters in final position signals that this is the end of the word, preventing addition errors. In LPD, final form letters might be taken to encode two types of information. They not only include letter identity information, but also contribute an additional source of information regarding letter position, and this might help in fixing the final letter in final position.

These results pertain to the status of the information concerning final forms in the process of letter identification. Where is this information encoded? Are final forms part of abstract letter identity, ALI? [1–3,6,15, 18,21] but see findings in [13] and review on the effect of case mixing on word familiarity; see [19] for a review.] The creation of abstract letter identity representations involves abstracting away from information that is irrelevant to word reading, such as information about fonts, size, case, or letter color. One possibility is that readers of Hebrew do not abstract away from final forms during the creation of ALI, and encode final forms in the ALI. Alternatively, if final forms are not encoded in the ALI itself, it might be that in addition to ALI that does not include letter forms, an additional (fourth) parallel encoding is created and used in word reading, that of final letters.

One further, perhaps more extreme possible interpretation of the data is that final-form letters and their corresponding middle-form counterparts actually have different abstract letter identity units. Taking Polk and Farah's [18] account for the way ALI is constructed in English, two letters are ascribed to the same ALI if they appear in the same context of other letters. However, in Hebrew this is never the case – each word either contains the letter in final form, if it is a final letter, or in middle form. There is no case in which final and middle forms interchange in a certain word, therefore, the system cannot decide that they are actually one and the same abstract letter, and might be forced to create two separate abstract letter units. Under this account, each of the two separate abstract representations of the form-changing letters has its own grapheme-to-phoneme conversion rules, which are identical in most cases.[2] This might be the reason why in LPD, for example, final letters do not move to other positions: because letter identification is unimpaired in LPD, and given that the final and middle forms are two different abstract letters, they cannot be interchanged in LPD.

In this respect, Arabic orthography is also interesting, because letters in Arabic have up to 5 different forms – initial, middle, final, connecting and not connecting to the previous letter. How would this affect migration errors in LPD? A study we recently conducted with a bilingual Arabic and Hebrew speaking individual with acquired Letter Position Dyslexia (and Letter Position Dysgraphia) suggests that letter forms constrain and reduce the number of migration errors in LPD in Arabic as well. The preliminary results from this patient show that he had more LPD errors in reading words with migration potential in Hebrew than in Arabic, and that out of the 10 migration errors he made in reading Arabic (out of 111 migratable words), only one was form-changing. Furthermore, it might be that the higher error rate he had in reading Hebrew (12.5% migration errors) than in reading Arabic (9% migrations) was due to the existence of letter forms in middle position in Arabic which assisted in preventing letter migrations. More data is required with respect to the effect of letter forms in Arabic on letter migrations.

The current findings regarding final-form letters in Hebrew open new questions regarding the applicability of the results to capital letters in languages like English, Italian and German. Like in Hebrew, capitals occur on the left side of words; unlike in Hebrew, their role is not merely orthographic. In English, Italian and other languages, capitals play two main roles: they mark the lexical category of single words: nouns in German, proper names in English, Italian and other languages, and they mark the beginning of sentences. A capital in the single word level carries a syntactic-semantic role: it identifies the lexical category of the word, and discriminates it from other lexical categories. A capital at the beginning of a sentence might also be used for the parsing of text. Therefore, for this information to

[2] Interestingly, for two of the five form-changing letters, (ף -פ; ך -כ), the grapheme-to-phoneme conversion rules are not identical for middle and final forms, as the middle form (without diacritics) is converted either to the plosive or to the fricative פ ⇒ /f, p/; כ ⇒ /x; k/, whereas the final form has only the fricative reading. When words borrowed from other languages which end with the plosive /p/, like /*tip*/, are written in Hebrew, the plosive is exceptionally written using the medial letter-form in final position.

be used in lexical processing, some information on the letter case should be encoded in these languages.

On the other hand, if we take the idea that when two forms interchange in the same position in the same word context they are ascribed the same abstract letter identity [18], then because every lower case initial letter can appear also as a capital letter, at the beginning of a sentence, the lower and upper case letters will be identified as two instances of the same ALI. This would allow the input of a word like *apricot*, and *Apricot* at the beginning of a sentence to access the same lexical item. These considerations suggest that capital letters are assigned the same ALI as their lower case counterparts, but there must be an additional encoding that signals that there was a capital letter, for the sake of syntactic-semantic processing.

The difference in the role of final-form letters and capital letters also reflects in the level of violation when an inappropriate form is used. In Hebrew, when a word ends in a middle-form letter (following neglect omission for example), this forms a violation of the orthographic well-formedness of the word, namely a violation that can be detected at the visual analysis level. Unlike Hebrew, in the case of English proper names and German nouns, when the word does not start in a capital this does not form a violation at the early orthographic level, but rather a violation at a later lexical stage. Thus, violations of final-forms in Hebrew would be ruled out already at the visual analysis system, but initial lower case letters in proper names or nouns will only be detected at the lexical stage.[3]

The question now is how these considerations would reflect in peripheral dyslexias in languages like English and German. If the level in which the violation of letter form is detected (orthographic or semantic) is the critical factor, and only violation at the orthographic level can be detected in peripheral dyslexia, then we would not expect errors in neglexia to be modulated by violations of capital letters, at least on the single word level. Similarly, if only different ALIs yield an effect on reading, then in German and English we would not expect sensitivity to capital letters. However, if the relevant factor is whether or not the information on capital letters is encoded, then English, Italian, and German-speaking individuals with left word-based neglexia are expected to be sensitive to initial letter case similarly to the sensitivity we reported for Hebrew-speaking individuals to final forms. In this respect two positions should be explored: sentence initial and word initial. If sensitivity to sentence initial capitals is preserved, less omissions of initial letters in the first word in the sentence compared to the rest of the words is expected. On the single word level, if, for example, German-speaking individuals with neglexia are sensitive to initial capital letters, they should be able to use capitals to tell nouns from homophonic verbs on the basis of the existence of initial upper case in noun-verb homophones such as Macht / macht, Schwamm / schwamm or Stand / stand; English-speaking individuals should be able to determine, when they read "*I saw Tom cruise alone*", whether any cruising has taken place, and should be sensitive to the differences between "*I hate to see William hurt*" and "*I like to see William Hurt*".

[3]Another relevant difference between capitals in languages like English and final forms in Hebrew is that capitals are not exclusive to initial position: While in Hebrew final letters appear exclusively in final position, capitals in English occur in medial position too – for example in acronyms and abbreviations (like BBC and LPD), and in some names (McDonald) (see [21] for a discussion of capital letters).

## Acknowledgements

We are grateful to Ivana Nachman-Katz for the data from the children with developmental neglect, and from some of the children with developmental LPD, Einav Rahamim for the data from children with developmental LPD, and to Julia Reznick for fruitful discussions of final letters. The research was supported by the joint German-Israeli BMBF-MOS Research Program Grant in Neuroscience GR01791 (Friedmann).

## Appendix: background details on the participants

Acquired neglexia

| Participant | Age | Gender | Word neglect | General neglect | Etiology |
|---|---|---|---|---|---|
| BR | 79 | female | left | | Right CVA- subacute infract in MCA |
| HZ | 43 | female | left | left | Right CVA hemorrhage-right basal and intraventricular |
| ZR | 60 | male | left | left | Right CVA |
| XM | 57 | male | left | | Right CVA-acute infarct internal capsule |
| BT | 65 | female | left | left | Right CVA |
| CL | 62 | male | left | left | Right CVA hemorrhage-right basal and intraventricular |
| MH | 58 | male | left | left | Thromboangiitis obliterans. Right internal capsule acute infarction and a lacunar infarct in the territory of the right vertebro-cerebrellar artery |

Developmental neglexia

| Participant | Age | Gender | Handedness | Grade | Class type |
|---|---|---|---|---|---|
| NT | 9:11 | male | right | 4 | small |
| IZ | 11:4 | male | right | 5 | small |
| VL | 9:1 | female | right | 4 | regular |
| SP | 9:8 | male | right | 5 | small |
| ID | 12:11 | male | right | 6 | small |
| SS | 12:6 | male | right | 6 | regular |
| OR | 10:3 | female | right | 4 | small |
| DN | 9:11 | male | right | 6 | small |
| ST | 10:3 | male | right | 6 | small |

Acquired LPD

| Participant | Age | Gender | Handedness | Etiology |
|---|---|---|---|---|
| BS | 75 | male | right | left parieto-occipital tumor removal craniotomy, hematoma at the bed of the tumor |
| PY | 70 | male | right | left ischemic parieto-occipital infarct |

Developmental LPD

| Participant | Age | Gender | Handedness | Grade | Class type |
|---|---|---|---|---|---|
| SP | 9:4 | female | right | 3 | small |
| YS | 29:10 | male | left | – | – |
| DV | 10:10 | male | right | 5 | regular |
| HN | 11:3 | male | right | 5 | small |
| SL | 9:11 | female | right | 4 | regular |
| SN | 11:2 | male | right | 5 | small |
| NS | 13:11 | female | right | 7 | regular |
| RM | 9:6 | male | right | 3 | small |
| RI | 15:9 | male | left | 10 | small |
| AN | 9:9 | female | right | 4 | regular |
| HA | 13:2 | female | right | 8 | LD school |
| ST | 11:5 | male | right | 5 | small |
| SR | 11:0 | female | right | 5 | small |
| RMI | 10:0 | male | right | 3 | small |
| SNI | 11:7 | male | right | 5 | small |
